# Translocation of an Intrauterine Contraceptive Device: Incidental Finding in the Rectosigmoid Colon

**DOI:** 10.1155/2010/404160

**Published:** 2010-06-09

**Authors:** R. Vilallonga, N. Rodriguez, M. Vilchez, M. Armengol

**Affiliations:** ^1^Servicio de Cirugía General y del Aparato Digestivo, Unidad de Cirugía Endocrina, Bariàtrica y metabólica, Hospital Vall d'Hebron, Universidad Autónoma de Barcelona, 08035 Barcelona, Spain; ^2^Servicio de Cirugía General y del Aparato Digestivo, Hospital Vall d'Hebron, Barcelona, Universidad Autónoma de Barcelona, 08035 Barcelona, Spain

## Abstract

The presence of an intrauterine device (IUD) within the colon is rare. Complications have been reported with IUDs among which uterine perforation. Translocation of IUDs to the uterine cavity, to the bladder and also through the wall of the bowel, and sigmoid colon has been reported. We believe there may be a case that surgeons should know the result of despite being a priori gynaecological complication. This paper reports on a case of colon perforation by an IUD.

The IUD is a commonly used reversible birth control method. One of the rare, but potentially serious complication is uterine perforation. In this paper, we report the exceptional case of an asymptomatic IUD translocation to the rectosigmoid colon lumen secondary to uterine perforation.

A 31-year-old patient was admitted to the emergency department complaining of proctalgia for one month. Unfortunately, no information was available regarding the IUD insertion procedure. The patient had a delivery fifteen months before. A rectal exploration showed the presence of a foreign body. An X-Ray showed a IUD. CT scan was performed and showed a normal uterus and a metallic piece entering the rectum. This CT scan incidentally revealed the presence of an IUD in the lumen of the rectosigmoid colon (See Figure 1). Removal of the device by means of endoscopic procedure was not performed. The patient underwent a surgical exploration and the IUD was removed from the rectum transanally. The postoperative course was uneventful.

Uterine perforation is a rarely observed complication. The incidence of IUD perforation ranges from 0.05/1,000 to 13/1,000 [1, 2]. Many authors have recommended that IUDs should be inserted by skilled providers to prevent complications such as uterine perforation [3]. IUD migration is more frequent in women who undergo labour with their IUD in place. In this last situation, due to the reduction in the size of the uterus and thinning of the uterine walls in the postpartum as a result of hypoestrogenemia, the uterus becomes more susceptible to perforation [2]. May be this could have contributed to the perforation in the case presented here.

May be this could have been another explanation in our case. Colon perforation is rare but has been described previously [4].

Another location of migration is the bladder because of its close proximity to the uterus [5] or the peritoneal cavity [6]. Other cases have been described as mimicking chronic appendicitis [7]. X-Ray and TVS can be helpful but CT scan will provide clear information about IUD location.

Another controversial issue is the treatment of cases of a perforated IUD. The general consensus is that the IUD should be removed to prevent infection, injury to the neighbouring organs, and intrabdominal adhesion formation. Fatal complications due to sepsis and intestinal obstruction have been described [1, 2]. However, copper IUDs perforated in the abdominal cavity rarely cause serious complications [8]. In case of perforation of the colon, there is a risk of fistula formation resulting in serious morbidity, treatment should not be differed [9]. Endoscopic treatment has been proposed and preferred. Also surgery can be required, and laparoscopy is frequently preferred [8].

Some authors have suggested leaving the IUD in place if the patient is asymptomatic as there may be less risk than performing a laparotomy or even a laparoscopy. However, it is up to the clinical judgment of the physician to decide on the preferred treatment strategy [3].

In conclusion, asymptomatic migration of IUD to the sigmoid colon lumen can occur. Skilful insertion is important to avoid complications. In case of perforation through the wall of the bowel, removal of the IUD is recommended because of the risk of fistula formation and colon perforation with a high ensuing morbidity. The specific type of the IUD should be known before deciding to remove the IUD or not. This case report highlights the need to conduct a follow-up examination after insertion of an IUD to verify the proper location of the IUD.

## Figures and Tables

**Figure 1:**
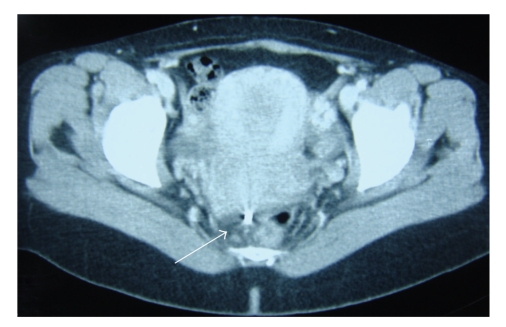
IUD is displayed in the rectum (arrow) after perforating the uterus located in front.
